# Polymyositis-Dermatomyositis and Interstitial Lung Disease in Pregnant Woman Successfully Treated with Cyclosporine and Tapered Steroid Therapy

**DOI:** 10.1155/2019/4914631

**Published:** 2019-03-11

**Authors:** Saito Mayu, Sakiko Isojima, Yoko Miura, Shinichiro Nishimi, Mika Hatano, Takahiro Tokunaga, Ryo Takahashi, Keiko Koide, Yusuke Miwa

**Affiliations:** ^1^Department of Rheumatology, Showa University School of Medicine, Tokyo, Japan; ^2^Division of Obstetrics and Gynecology, Showa University School of Medicine, Tokyo, Japan

## Abstract

Polymyositis-dermatomyositis is extremely rare during pregnancy, and immunosuppressive therapy should be administered after carefully considering the effects on both the mother and fetus. Several reports have associated the disease activity with fetal prognosis, higher rates of eclampsia, preterm births, and fetal deaths. We report our experience with a patient who was diagnosed with polymyositis-dermatomyositis complicated by interstitial lung disease during pregnancy and was treated with a combination-immunosuppressant regimen. To the best of our knowledge, this is the first case wherein cyclosporine was used concomitantly with a steroid for the treatment of polymyositis diagnosed during pregnancy, with successful outcome of childbirth without any complications.

## 1. Introduction

Polymyositis-dermatomyositis is an autoimmune inflammatory myopathy that affects the proximal muscles. In 40% of the cases, interstitial lung disease, which is one of the negative prognostic factors that is associated with morbidity and mortality, is complicated. It is common that the onset in childhood (5–15 years) and middle age (40–60 years), therefore, unlike systemic lupus erythematosus, polymyositis-dermatomyositis which pregnant women are complicated with is extremely rare. Furthermore, immunosuppressive therapy should be administered with careful consideration about the effects on both the patient and her fetus. We report a case wherein cyclosporine was used concomitantly with a steroid for the treatment of polymyositis diagnosed during pregnancy, with successful outcome of childbirth.

## 2. Case Presentation

A 27-year-old woman was referred to our department upon suspicion of polymyositis-dermatomyositis from the division of Endocrinology and Metabolism. One year ago, she had a stillbirth at 21 weeks of gestation. Blood tests performed at the time revealed a marked increase in creatine phosphokinase (CK) levels, and she had experienced symptoms of muscle weakness and exertional dyspnea since the discharge, but no detailed examination was performed. She became pregnant again, and her exertional dyspnea worsened at 8 weeks of gestation. She was administered insulin therapy for glucose metabolism disorder diagnosed in the first trimester by an obstetrician in the division of Endocrinology and Metabolism. As her muscle enzyme levels were relatively higher than those recorded in the previous year, she was suspected to have an autoimmune myositis (myopathy) and was referred to the division of Rheumatology and admitted to our hospital at 15 weeks of gestation.

**Figure 1:**
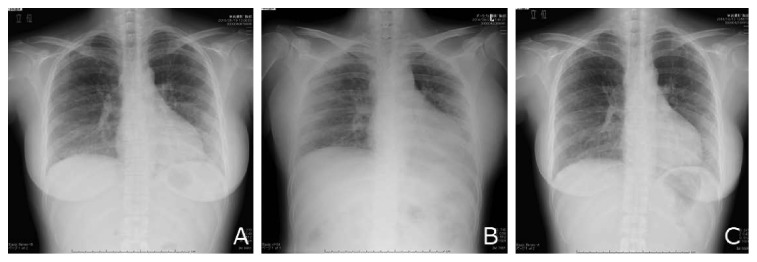
(a) Chest radiography on admission showing bilateral ground-glass opacity, (b) discharge radiography showing improvement of ground-glass opacity, and (c) postpartum radiography showing more improvement.

**Figure 2:**
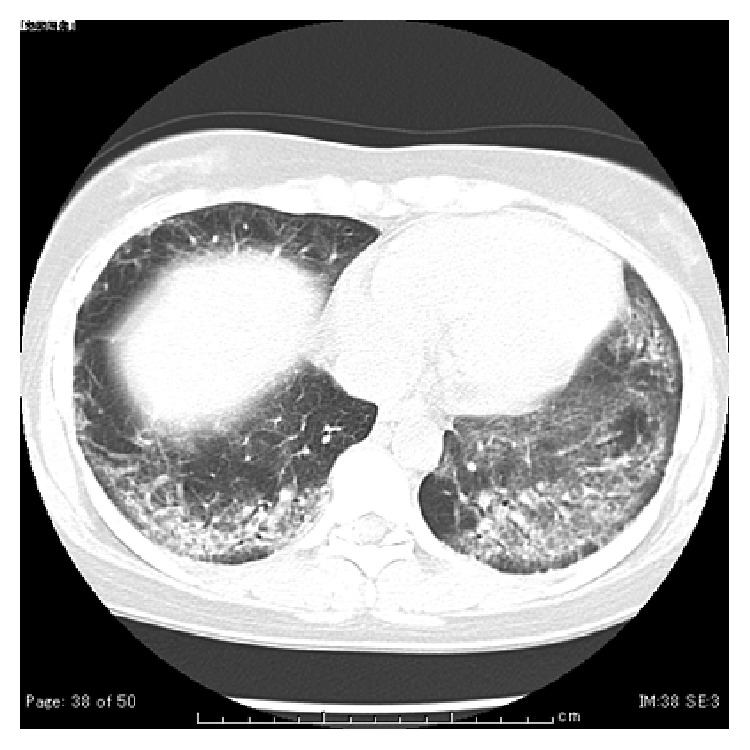
Chest-computed tomography on admission showing ground-glass opacity with partial honeycomb lung throughout the entire lung field.

She had elevated serum muscle enzymes (CK 3875 U/L, aldolase 29.5 U/L, and myoglobin 440 ng/mL), myalgia, muscle weakness, and arthritis; further, she showed myogenic change on electromyogram, inflammatory reaction, tested positive for anti-Jo1 antibodies (32-fold higher than normal), but no skin lesion; therefore, she was diagnosed with polymyositis based on Bohan's criteria [1]. Furthermore, she had exertional dyspnea, and the ambulatory SpO_2_ decreased to 84% (at room atmosphere), and blood tests revealed elevated interstitial lung disease marker levels (KL-6: 1572.0 U/mL; SPD: 195.5 ng/mL). We explained the risks associated with fetal exposure to radiation to the patient, and with her consent, we acquired a chest radiograph (Figure 1(a)) and performed chest-computed tomography (Figure 2) and also conducted a respiratory function test. We observed ground-glass opacity with a partial honeycomb lung throughout the entire lung field and restrictive respiratory impairment of 46.0% in 1 s; thus, she was diagnosed with interstitial lung disease.

We explained to the patient the necessity for immediate initiation of steroid therapy and concomitant use of immunosuppressant as needed. We also explained that she might be difficult to tolerate labor if her respiratory condition worsened and have pregnancy complications including intrauterine growth restriction that was due to mother's hypoxia or immunosuppressive therapy and preterm birth; however, she and her family hoped to continue the pregnancy.

**Figure 3:**
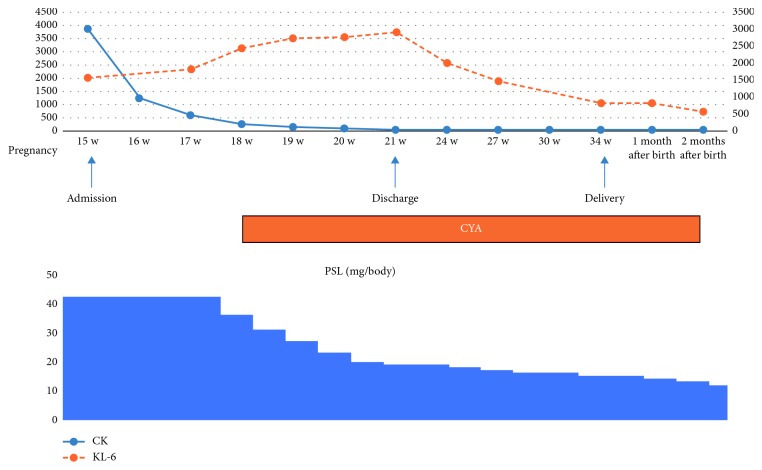
After starting steroid therapy (prednisolone 42 mg/day), serum muscle enzyme levels were decreasing, but interstitial lung disease marker levels were increasing. So, administration of concomitant cyclosporine was started at 18 weeks of gestation. Prednisolone dose was decreased at a rate of 15%/week. She was discharged at 22 weeks and delivered at 34 weeks and 1 day of gestation.

Administration of prednisolone (PSL) 42 mg/day (0.8 mg/kg) was started on the day of admission. 1 L/min oxygen therapy was required against exertional dyspnea. In management of glucose metabolism disorder, intensive insulin therapy was needed at the same time when high-dose steroid therapy was provided. After starting the therapy, muscle weakness improved rapidly and serum muscle enzyme levels were decreasing (Figure 3). However, her symptom of exertional dyspnea did not significantly improve, and interstitial lung disease marker levels were increasing. Considering the risk of complications such as hypertensive disorders of pregnancy, infections aggravated by high-dose steroid therapy, and the risk of exacerbation of disease due to steroid tapering, administration of concomitant cyclosporine (CYA) 200 mg/day was started at 18 weeks of gestation.

After completion of high-dose steroid therapy, the PSL dose was decreased at a rate of 15%/week, and the CYA blood level was monitored by setting peak levels of 1500–2000 ng/ml and trough levels of 100–150 ng/ml before increasing the dose to 275 mg/day at 21 weeks of gestation. At that time, her dyspnea got improved, and interstitial lung disease marker levels decreased; further, oxygen therapy was discontinued at the time of discharge. After high-dose steroid therapy, a chest radiograph (Figures 1(b) and 1(c)) showed improvement of radiological signs. An obstetrician regularly performed a fetal monitoring with ultrasound during hospitalization. Though fetal growth restriction (−1.6SD) was diagnosed with ultrasound at 22 weeks of gestation, the obstetrician decided to manage the fetus in the outpatient clinic, so that there was no any other signs suspecting uteroplacental dysfunction. The patient was discharged at 22 weeks of gestation.

Her steroid dose had been tapered in the outpatient clinic; however, her blood pressure increased and absence of end-diastolic flow velocity in the umbilical artery was observed at 34 weeks of gestation; therefore, she resulted in termination of pregnancy at 34 weeks and 1 day of gestation. She delivered a male baby by cesarean section, and the birth weight was 1,594 g, which was light for date, and the Apgar score was 8/9. The baby was hospitalized in the neonatal intensive care unit. The baby showed no obvious adverse effects of preterm birth, except hypoglycemia prolonged until Day 1. He was discharged at Day 38. The patient has undergone steroid tapering therapy as an outpatient even after the delivery, and her disease remains under stable condition.

## 3. Discussion

In the present case, it is supported that she already got polymyositis-dermatomyositis before she became pregnant, and it was aggravated by pregnancy. Katz [2] and Morihara et al. [3] have described a relationship between autoimmune myositis (myopathy) and pregnancy as attributable to changes in the mother's hormones or the fetus being recognized as a foreign body. Missumi et al. [4] reported in their case series covering 15 cases that the disease activity of myositis does not increase because of pregnancy; however, several other reports have associated the disease activity of myositis with fetal prognosis, and it has, moreover, been associated with higher rates of preeclampsia, preterm birth, or fetal death [5–7]. According to these reports, favorable control of disease activity before or during pregnancy would likely improve the prognosis of both the mother and child in pregnancies complicated with autoimmune myositis (myopathy). In the present case, the time when the patient was diagnosed was 15 weeks of gestation, and because of a preexisting complication of severe interstitial lung disease, the child was at risk of exposure to hypoxia and strong immunosuppressive therapy. Furthermore, the mother was at risk of complications such as hypertensive disorders of pregnancy, glucose metabolism disorder induced by steroids, and respiratory impairment during labor. In previous reports, autoimmune myositis (myopathy) complicated with interstitial pneumonia during pregnancy is extremely rare. In a case series of 9 cases reported by Vancsa et al. [8], one case was complicated with interstitial lung disease, underwent steroid pulse therapy and maintenance therapy by internal administration of steroids, and delivered a healthy baby weighing 1,680 g at 35 weeks of gestation. Ochiai et al. [9] reported that they administered high-dose steroid therapy to a pregnant woman with clinically amyopathic dermatomyositis, complicated with progressive interstitial lung disease, developed during pregnancy. This patient delivered a healthy baby weighing 2,147 g at 34 weeks and 5 days of gestation, and disease activity was well controlled during pregnancy.

In the present case, we gave the first priority to controlling the activity of interstitial lung disease and added CYA to the high-dose steroid therapy at 18 weeks of gestation. In earlier reports, steroids, immunoglobulins, and hydroxychloroquine were selected as therapies for pregnant women with myositis or interstitial lung disease [10–12]. We did not select other treatments such as increased dose of steroids owing to the risk of exacerbation of hypertensive disorders of pregnancy and glucose metabolism disorder. And we did not also select immunoglobulins because of insufficient evidence for the treatment of interstitial lung disease of inflammatory myositis. The Guidelines for Obstetrical Practice in Japan 2017 recommend CYA as a treatment for pregnant women complicated with diseases such as steroid-resistant systemic lupus erythematosus. However, to our knowledge, this is the first report about concomitantly use of CYA with a steroid for pregnant woman complicated with autoimmune myositis (myopathy).

More than 800 pregnant women receiving CYA have been reported, mainly in transplant recipients [13]. The observed rate of congenital malformations has not exceeded than that which is reported in the general population. But it is unclear whether or not CYA administered to mother is associated with fetal prematurity and low birth weight. In addition, Stanley et al. reported that a delay in mental development was observed in 16% of children exposed to CYA in utero [14]. However, we thought that the advantage of using CYA for maternal treatment was greater. We believe that tapering dose of steroid smoothly with CYA use contributed to the following: (1) attenuation of pregnancy complications such as hypertensive disorders or pregnancy glucose metabolism disorder that may be triggered by steroid therapy and (2) prevention of disease exacerbation. Like steroids, CYA causes side effects such as hypertension, but she did not require antihypertensive drugs.

We report that achieving control of disease activity before delivery and minimizing steroid dose are considered to contribute to favorable outcomes for both the mother and her baby. CYA was approved as a drug for pregnant women in Japan in July 2018, so CYA will be used for the patients such as our case in the future.

## Additional Points

We may be able to effectively and safely use cyclosporine for the mother and the fetus in the treatment of autoimmune disease in pregnancy when disease activity cannot be controlled by merely using steroids.
